# Genome sequences of the Shiga-like toxin-producing *Escherichia coli* NCCP15655 and NCCP15656

**DOI:** 10.1186/s13099-015-0060-6

**Published:** 2015-05-12

**Authors:** Min-Jung Kwak, Soon-Kyeong Kwon, Seung-Hak Cho, Jihyun F Kim

**Affiliations:** Department of Systems Biology and Division of Life Sciences, Yonsei University, 50 Yonsei-ro, Seodaemun-gu, Seoul, 120-749, Republic of Korea; Biosystems and Bioengineering Program, University of Science and Technology, 217 Gajung-ro, Yuseong-gu, Daejeon, 305-350, Republic of Korea; Division of Enteric Diseases, Center for Infectious Diseases, Korea National Institute of Health, Heungdeok-Gu, Cheongju, 363-951 Republic of Korea

**Keywords:** Pathogenic *E. coli*, Shiga toxin, Verotoxin, Hemolytic uremic syndrome, Hemolysin

## Abstract

**Background:**

Virulence genes can spread among commensal bacteria through horizontal gene transfer. The bacterium with novel virulence factors may pose a severe threat to public health because of the absence of a management system unlike known pathogens. Especially, when a pathogenic bacterium acquires a new kind of virulence genes, it tends to exhibit stronger virulence. In this study, we analyzed the genomes of the two strains of *Escherichia coli* that were isolated from the feces of patients with diarrhea and produce Shiga-like toxin.

**Results:**

Phylogenetic analysis of conserved genes and average nucleotide identity values of the draft genome sequences indicate that strains NCCP15655 and NCCP15656, isolated from diarrhea patients, belong to the B1 group of *E. coli* and form a sister clade with strain E24377A. However, the proportion the genes belonging to the subsystem category “phages, prophages, transposable elements, plasmids” and “virulence, disease and defense” are higher than E24377A. Indeed, in their genomes, genes encoding Shiga toxin type 1, Shiga toxin type 2, and type 1 fimbriae were detected. Moreover, a plasmid encoding hemolysin and entropathogenic *E. coli* secreted protein C was identified in both genomes.

**Conclusions:**

Through the genome analysis of NCCP15655 and NCCP15656, we identified two types of Shiga-like toxin genes that could be responsible for the manifestation of the diarrhea symptom. However, the LEE island, which is one of the major virulence factors of enterohemorrhagic *E. coli*, was not detected and they are most similar with non-Shiga-like toxin-producing *E. coli* at the genomic level. NCCP15655 and NCCP15656 will be good examples of Shiga-like toxin-producing *E. coli* whose genomes are not as similar with typical enterohemorrhagic *E. coli* as non-Shiga-like toxin-producing *E. coli*.

## Background

Shiga-like toxin-producing *Escherichia coli* (STEC), also called verotoxin-producing *E. coli*, is a major pathogenic group of *E. coli* that causes bloody diarrhea and hemolytic uremic syndrome (HUS) and enterohemorrhagic *E. coli* (EHEC) is one of such STEC [1]. Gene(s) encoding the Shiga-like toxin (Stx) are carried by a lambdoid phage and the most frequently isolated serotypes of Shiga-like toxin-producing EHEC are O157, O104, O26, O111, and O145 [1-3]. *E. coli* is a common member of the normal flora in the large intestine, but sometimes they acquire pathogenic genes from other bacteria or bacteriophages. Indeed, there are several cases in which non-pathogenic strains or unknown serotypes of STEC cause diseases with symptoms similar to those of the STEC strains [4-6]. The causative organism of the 2011 German outbreak, which is the largest STEC outbreak [7,8], is *E. coli* O104:H4 that is enteroaggregative *E. coli* (EAEC) harboring the Stx prophage [9]. The major virulence feature of EHEC is the Shiga-like toxin, which is an exotoxin that causes cellular toxicity. Another feature of EHEC is intimin, which is an outer-membrane adhesion protein encoded by the locus for enterocyte effacement (LEE) island [10]. The major virulence factor of EAEC is aggregative adhesion fimbriae, which mediate bacterial adherence and make ‘stacked brick wall’ structure on the host cells [11]. This EHEC/EAEC hybrid strain also acquired plasmid-encoded antibiotic resistance genes and exhibited strong virulence [12]. In South Korea in 2002 and 2006, there were two case reports that the serotype O8 and O104:H4 *E. coli* strains caused HUS in a 16 year-old man [13] and a 29 year old woman [14], respectively. Moreover, in 2012, we reported the genome sequence and analysis results of the virulence genes of EHEC strains isolated from Korea [2,3,15]. To reveal the genomic features of STEC in Korea, we sequenced a dozen of *E. coli* strains from diarrhea patients in Korea from 2001 to 2011. Among them, two strains of Shiga-like toxin-producing *E. coli* belonging to same group were selected for genome analysis. In this study, we reported the genomes of two *E. coli* strains, named as NCCP15655 and NCCP15656, which had been isolated from the feces of a female patient and a male patient with diarrhea in South Korea in 2003. In the strains, the gene encoding Shiga-like toxin was detected, but serotypes were not determined by experiment. Through the genome analysis of these two isolates, we report a case of pathogenic *E. coli* strains with two types of Shiga-like toxin genes in a single genome whose structure is most similar to non-EHEC strains.

## Methods

### Bacteria and DNA isolation

In 2003, two *E. coli* strains were isolated from stool samples of a female patient and a male patient with symptom of diarrhea in Korea. To test the presence of the Shiga-like toxin genes (*stx*1 and *stx*2), the two strains were subject to PCR with the primers specific to *stx*1 (F′-CGTACGGGGATGCAGATAAATCGC and R′-CAGTCATTACATAAGAACGCCCAC) and *stx*2 (F′-GTTCTGCGTTTTGTCACTGTCAC and R′-GTCGCCAGTTATCTGACATTCTGG). These two strains were deposited at the National Culture Collection for Pathogens in Korea National Institute of Health (KNIH) and their accession numbers are NCCP15655 (from a female patient) and NCCP15656 (from a male patient). Genomic DNA was extracted using chemical and enzymatic methods as described in Molecular Cloning, A Laboratory Manual [16].

### Genome sequencing, assembly and annotation

Genome Analyzer IIx of the Illumina-Solexa platform at the Biomedical Genomics Research Center of Korea Research Institute of Bioscience and Biotechnology was used for genome sequencing. 22,525,438 high-quality reads with 233-fold coverage for NCCP15655 and 27,858,714 high-quality reads with 235-fold coverage for NCCP15656 were generated from 500-bp paired-end libraries. Sequence trimming and *de novo* assembly were performed using CLC Genomics Workbench version 5.1 (CLC bio, Inc.) and scaffolding was carried out with SSPACE [17]. Automatic gap filling was performed using IMAGE [18] and manual gap filling was performed using CLC Genomics Workbench. Structural gene prediction was performed using Glimmer 3 [19] and functional annotation was performed using blastp against MicroScope database [20] of *E. coli* and *Shigella* species. We then employed automatic annotation using the RAST server [21] and compared it with the annotation result from MicroScope database for more accurate functional assignment. We also performed additional blastp against the subsystem database of the RAST server for the gene categorization.

### Gene clustering and phylogenetic tree construction

Core gene set of 71 genomes (60 *E. coli* strains, 10 *Shigella* strains, and 1 *Escherichia fergusonii*) was identified using OrthoMCL (version 2.0.3) [22] with parameters of *e*-value ≤ 1E-5, identity ≥ 85%, and coverage ≥ 80% [23]. Duplicated genes were excluded from the core gene set. 1,273 core genes were used for the phylogenetic tree construction. Amino-acid sequences of each core gene were aligned with MUSCLE (version 3.6) [24] and converted to phylip format after concatenation of all core genes. A maximum likelihood tree was constructed using PhyML (version 2.4.5) [25] with JTT evolutionary model [26].

### Other computational analysis

Average nucleotide identity values based on BLAST (ANIb) [27] were calculated by Jspecies [28] with ANI calculation parameters of identity ≥ 30% and coverage ≥ 70%. Clustered regularly interspaced short palindromic repeat (CRISPR) was detected with CRISPRfinder (http://crispr.u-psud.fr/Server/). Homology searches were conducted using the BLAST software. Serotype analysis was performed using SerotypeFinder (ver.1.0) in the center for genomic epidemiology server (https://cge.cbs.dtu.dk/services/). Subtype analysis of the *stx* genes was conducted with the sequence-based protocol [29].

## Quality assurance

Genome sequencing was conducted using a single bacterial isolate and contamination possibility was checked using CLC Genomics Workbench in the step of *de novo* assembly, mapping reads to contigs and generation of detailed mapping report. The contamination of other genomes can be checked through confirmation of coverage level distribution in a detailed mapping report as well as inspection of the alignment result with accurate paired distance.

## Initial findings

### Genome structure

The draft genome of *Escherichia coli* NCCP15655 and NCCP15656 consist of five contigs and 15 contigs, respectively. The sum of five contigs of NCCP15655 is 4,965,708 bp (50.86% G + C content) and 4,970 coding sequences (CDSs), seven ribosomal RNA operons and 97 tRNAs were predicted. The sum of 15 contigs of NCCP15656 is 4,925,312 bp (50.93% G + C content) and 4.919 CDSs, seven ribosomal RNA operons and 92 tRNAs were detected. NCCP15655 and NCCP15656 have two CRISPRs in each that consist of direct repeat sequences and seven spacer sequences. The spacers 5 and 6 in CRISPR 1 and spacer 7 in CRISPR 2 had no homology with sequences in the GenBank database.

### Phylogenetic relationship and comparison with closely related strains

**Figure 1:**
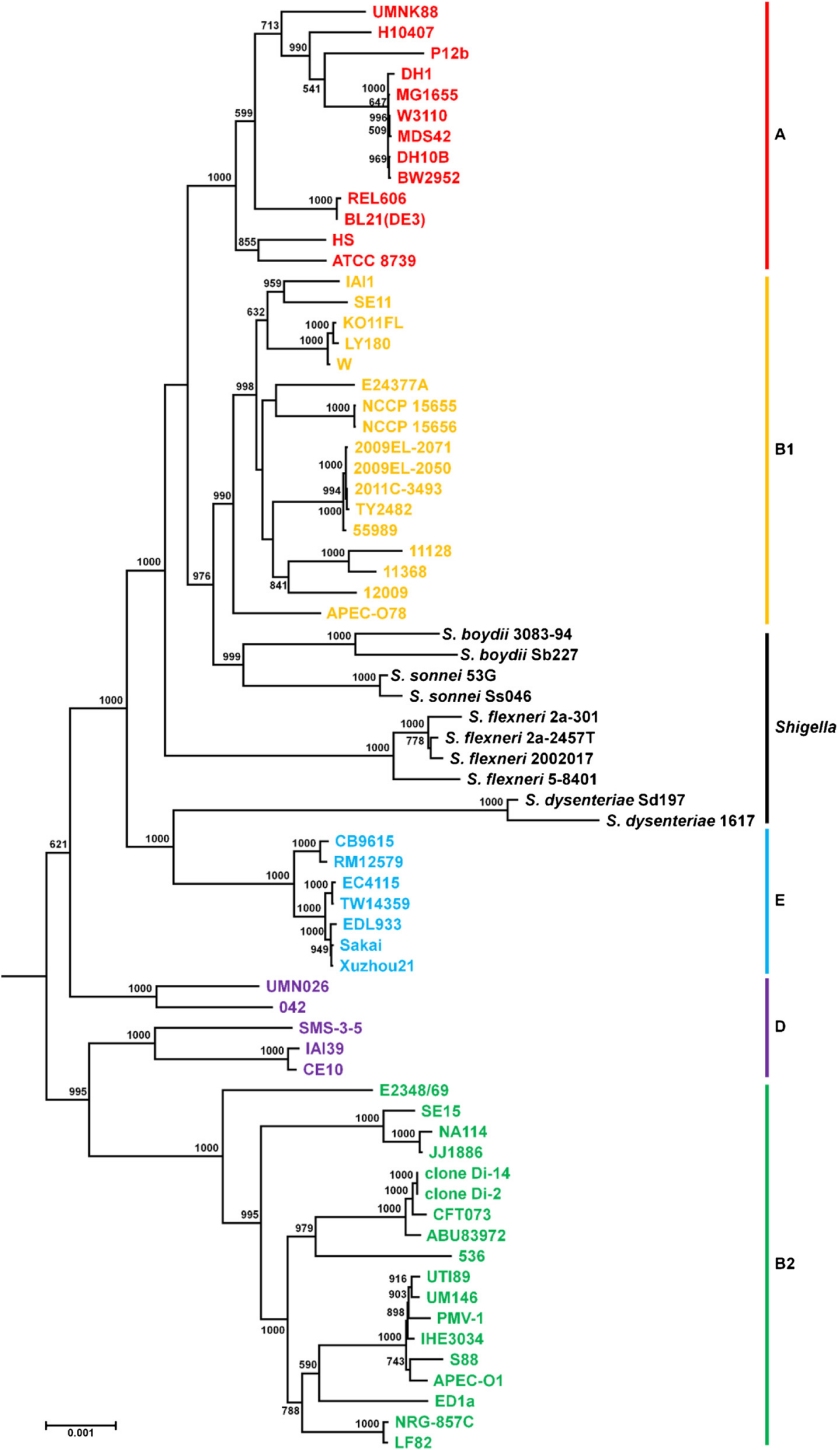
Phylogenetic relationship among genome-sequenced *E. coli* and *Shigella* strains. The phylogenetic tree was generated by PhyML with amino-acid sequences of 1,273 core genes from completely sequenced *E. coli* and *Shigella* strains. Each color indicates the phylogenetic group of *E. coli* (red, A; yellow, B1; black, *Shigella*; blue, E; purple, D; green, B2). Bootstrap values (percentages of 1,000 replications) greater than 50% are shown at each node. *Escherichia fergusonii* ATCC 35469 were used for the out-group. The scale bar represents 0.001 nucleotide substitutions per site.

**Figure 2:**
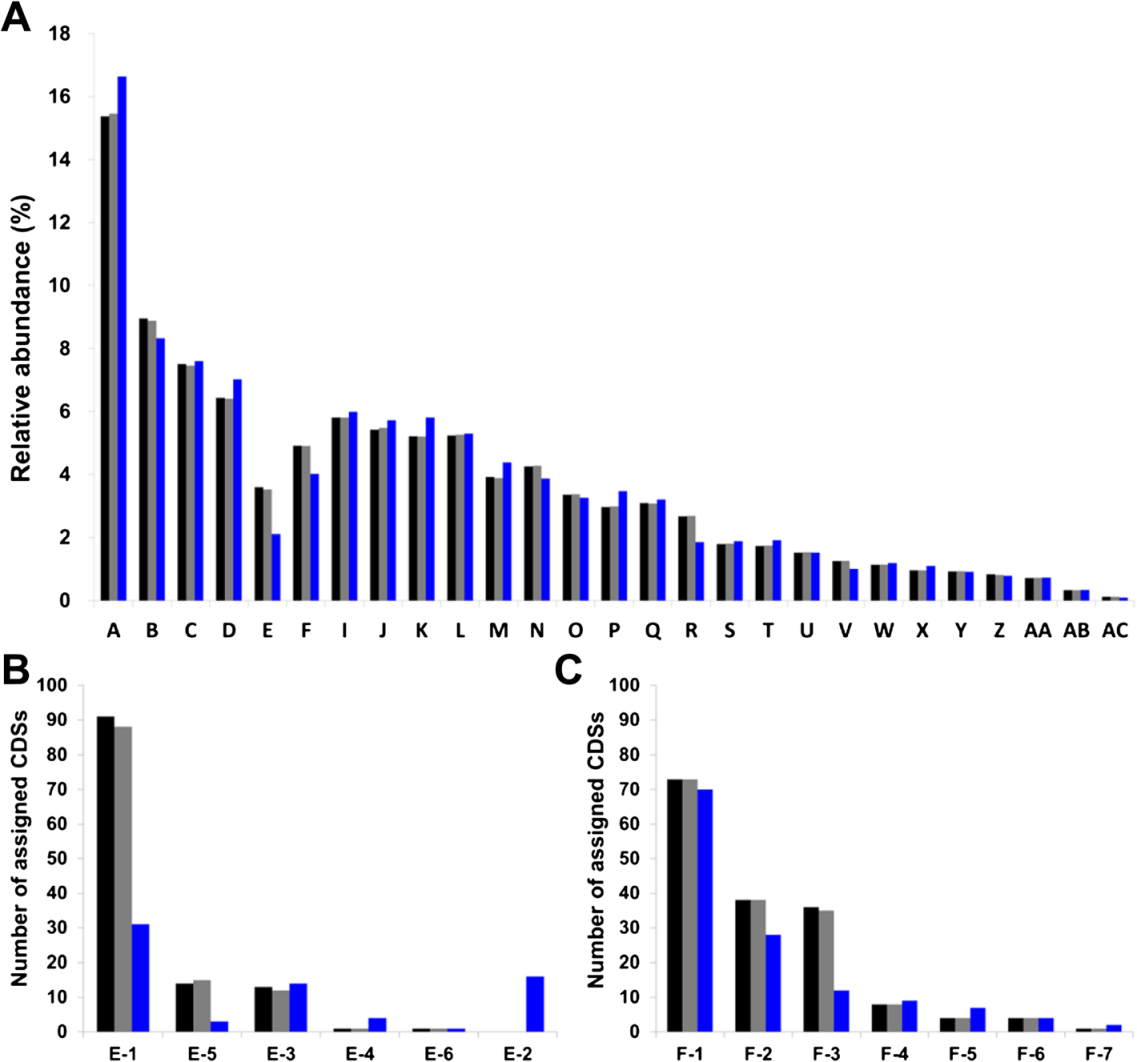
Comparison of the subsystem categories. Comparison results of the subsystem-assigned genes among NCCP15655, NCCP15656, and E24377A. **(A)** Relative abundance of the subsystem-assigned genes. A, Carbohydrates; B, Clustering-based subsystems; C, Amino acids and derivatives; D, Cell wall and capsule; E, Phages, prophages, transposable elements, plasmids; F, Virulence, disease and defense; I, Membrane transport; J, Protein metabolism; K, Cofactors, vitamins, prosthetic groups, pigments; L, Stress response; M, DNA metabolism; N, Respiration; O, Nucleosides and nucleotides; P, Regulation and cell signaling; Q, RNA metabolism; R, Motility and chemotaxis; S, Nitrogen metabolism; T, Fatty acids, lipids, and isoprenoids; U, Miscellaneous; V, Metabolism of aromatic compounds; W, Phosphorus metabolism; X, Cell division and cell cycle; Y, Iron acquisition and metabolism; Z, Sulfur metabolism; AA, Potassium metabolism; AB, Secondary metabolism; AC, Dormancy and sporulation. **(B)** Number of CDSs assigned to the sub-category of “Phages, prophages, transposable elements, plasmids”. E-1, Phages, prophages; E-5, Bacteriophage structural proteins; E-3, Bacteriophage integration/excision/lysogeny; E-4, Phage host interactions; E-6, Superinfection exclusion; E-2, Transposable elements. **(C)** Number of CDSs assigned to the sub-category of “Virulence, disease and defense”. F-1, Resistance to antibiotics and toxic compounds; F-2, Adhesion; F-3, Type III, type IV, type VI, ESAT secretion systems; F-4, Invasion and intracellular resistance; F-5, Fimbriae of the chaperone/usher assembly pathway; F-6, Bacteriocins, ribosomally synthesized antibacterial peptides; F-7, Toxins and superantigens. Bars: black, NCCP15655; gray, NCCP15656; blue, E24377A.

A phylogenomic tree was constructed using 1,273 core genes of NCCP15655, NCCP15656, and the completely sequenced strains in *Escherichia*/*Shigella* group. The tree showed that NCCP15655 and NCCP15656 belong to the group B1 and formed a sister clade with strain E24377A, which is an enterotoxigenic *E. coli* (ETEC) (Figure 1). ANIb values between strain NCCP15655/NCCP15656 and other strains belonging to B1 group were 98.27 ~ 99.08 (Table 1). NCCP15655 and NCCP15656 are Shiga-like toxin producing *E. coli* but they form a sister clade with ETEC strain E24377A despite of highest similarity of ANI value with non-pathogenic strains. Thus, we compared the genomic features using subsystem classification between NCCP1565/NCCP15656 and E24377A. In spite of the high similarity of genomes and phylogenetic proximity, there are distinct differences between NCCP15655/NCCP15656 and E24377A in the proportion of subsystem-assigned genes. Subsystem classification results showed that the proportions of the genes belonging to the subsystem category “phages, prophages, transposable elements, plasmids” and “virulence, disease and defense” are higher in NCCP15655 and NCCP15656 than E24377A (Figure 2 and Table 2). The number of genes belonging to the sub-category ‘phages, prophages’ and ‘bacteriophage structural proteins’ of “phages, prophages, transposable elements, plasmids” and sub-category ‘resistance to antibiotics and toxic compounds’, ‘adhesion’, and ‘type III, type IV, type VI, ESAT secretion systems’ of “virulence, disease and defense” are higher in NCCP15655 and NCCP15656 than E24377A. In the genome of NCCP15655 and NCCP15656, the genes belonging to sub-category ‘phages, prophages’ and ‘bacteriophage structural proteins’ include Stx phage and the genes belonging to sub-category ‘type III, type IV, type VI, ESAT secretion systems’ encoded conjugative plasmid-related proteins. A conjugative plasmid in NCCP15655 and NCCP15656 harbors the *hlyABCD* genes that encode a hemolysin.

**Table 1:** **Average nucleotide identity values based on BLAST between the completely sequenced members of the** ***E. coli*** **B1 group**

	**A**	**B**	**C**	**D**	**E**	**F**	**G**	**H**	**I**	**J**	**K**	**L**	**M**	**N**	**O**	**P**
**A**	---	99.98	98.9	98.93	98.86	98.79	98.89	98.83	98.86	98.87	98.79	98.81	98.85	98.84	98.64	98.67
**B**	99.99	---	98.9	98.94	98.87	98.8	98.88	98.85	98.87	98.91	98.85	98.87	98.9	98.84	98.67	98.7
**C**	99.08	99.08	---	99.23	99.89	99.88	99.07	98.92	99.06	98.91	99.01	99.03	99.02	98.99	98.85	98.94
**D**	99.05	99.04	99.19	---	99.15	99.09	99.22	98.98	99.13	99.11	99.11	99.12	99.11	99.08	98.9	99.02
**E**	99.04	99.04	99.92	99.17	---	99.87	99.09	98.94	99.05	98.98	99.04	99.05	99.03	99.03	98.89	99
**F**	98.94	98.94	99.86	99.1	99.92	---	98.96	98.78	98.93	98.82	98.89	98.89	98.89	98.89	98.76	98.86
**G**	98.88	98.86	98.94	99.11	98.95	98.87	---	98.84	98.93	98.95	98.88	98.93	98.93	98.93	98.6	98.78
**H**	98.84	98.83	98.97	98.99	98.94	98.85	98.92	---	98.91	98.87	98.83	98.82	98.83	98.82	98.8	98.91
**I**	98.83	98.83	98.95	99.11	98.99	98.88	98.89	98.83	---	98.91	98.91	98.95	98.94	98.86	98.66	98.83
**J**	98.81	98.79	98.81	98.96	98.81	98.7	98.83	98.76	98.82	---	99.8	99.79	99.81	98.73	98.6	98.8
**K**	98.79	98.8	98.84	98.98	98.82	98.75	98.81	98.74	98.84	99.79	---	99.96	99.96	98.77	98.58	98.72
**L**	98.76	98.76	98.81	98.92	98.8	98.7	98.81	98.68	98.73	99.78	99.93	---	99.96	98.69	98.54	98.73
**M**	98.75	98.75	98.78	98.9	98.76	98.65	98.78	98.67	98.74	99.76	99.89	99.93	---	98.68	98.49	98.73
**N**	98.58	98.58	98.6	98.64	98.58	98.49	98.43	98.72	98.75	98.62	98.53	98.54	98.65	---	98.61	98.8
**O**	98.43	98.47	98.53	98.59	98.55	98.46	98.27	98.6	98.71	98.58	98.46	98.45	98.57	98.73	---	99.27
**P**	98.27	98.33	98.44	98.57	98.47	98.34	98.23	98.64	98.56	98.5	98.34	98.39	98.54	98.7	98.99	---

A, NCCP15655; B, NCCP15656; C, W; D, IAI1; E, LY180; F, KO11FL; G, SE11; H, APEC O78; I, E24377A; J, 55989; K, 2011C-3493; L, 2009EL-2050; M, 2009EL-2071; N, 12009; O, 11128; P, 11368.

**Table 2:** **Number of the subsystem-assigned CDSs**

	**NCCP15655**	**NCCP15656**	**E24377A**
**Carbohydrates**	514 (15.38)	514 (15.46)	547 (16.64)
**Clustering-based subsystems**	299 (8.95)	295 (8.87)	274 (8.33)
**Amino acids and derivatives**	251 (7.51)	248 (7.46)	250 (7.60)
**Cell wall and capsule**	215 (6.43)	213 (6.41)	231 (7.03)
**Phages, prophages, transposable elements, plasmids**	120 (3.59)	117 (3.52)	69 (2.10)
**Virulence, disease and defense**	164 (4.91)	163 (4.90)	132 (4.01)
**Membrane transport**	194 (5.80)	193 (5.81)	197 (5.99)
**Protein metabolism**	181 (5.42)	182 (5.48)	188 (5.72)
**Cofactors, vitamins, prosthetic groups, pigments**	174 (5.21)	173 (5.20)	191 (5.81)
**Stress response**	175 (5.24)	175 (5.26)	174 (5.29)
**DNA metabolism**	131 (3.92)	129 (3.88)	144 (4.38)
**Respiration**	142 (4.25)	142 (4.27)	127 (3.86)
**Nucleosides and nucleotides**	112 (3.35)	112 (3.37)	107 (3.25)
**Regulation and cell signaling**	99 (2.96)	99 (2.98)	114 (3.47)
**RNA metabolism**	103 (3.08)	102 (3.07)	105 (3.19)
**Motility and chemotaxis**	89 (2.66)	89 (2.68)	61 (1.86)
**Nitrogen metabolism**	60 (1.80)	60 (1.81)	62 (1.89)
**Fatty acids, lipids, and isoprenoids**	58 (1.74)	58 (1.74)	63 (1.92)
**Miscellaneous**	51 (1.53)	51 (1.53)	50 (1.52)
**Metabolism of aromatic compounds**	42 (1.26)	42 (1.26)	33 (1.00)
**Phosphorus metabolism**	38 (1.14)	38 (1.14)	39 (1.19)
**Cell division and cell cycle**	32 (0.96)	32 (0.96)	36 (1.09)
**Iron acquisition and metabolism**	31 (0.93)	31 (0.93)	30 (0.91)
**Sulfur metabolism**	28 (0.84)	27 (0.81)	26 (0.79)
**Potassium metabolism**	24 (0.72)	24 (0.72)	24 (0.73)
**Secondary metabolism**	11 (0.33)	11 (0.33)	11 (0.33)
**Dormancy and sporulation**	4 (0.12)	4 (0.12)	3 (0.09)
**Subsystem-assigned CDSs**	3,342 (100.00)	3,324 (100.00)	3,288 (100.00)

Numbers in parentheses indicate the percentage of the subsystem-assigned CDSs.

Interestingly, although the two strains have been isolated independently from different individuals, the two strains are remarkably similar. In fact, the serotype determined by the *wzt* and *wzm* gene for O-antigen and the *fliC* gene for H-antigen indicated that the serotype of NCCP15655 and NCCP15656 is O8:H49. Moreover, at the genomic level, two strains are highly similar and ANIb values between the strains range from 99.98 to 99.99 (Table 1). Based on these relationships, we postulate that they might share a very recent common ancestor, if not clonal.

### Shiga-like toxin and virulence genes

**Figure 3:**
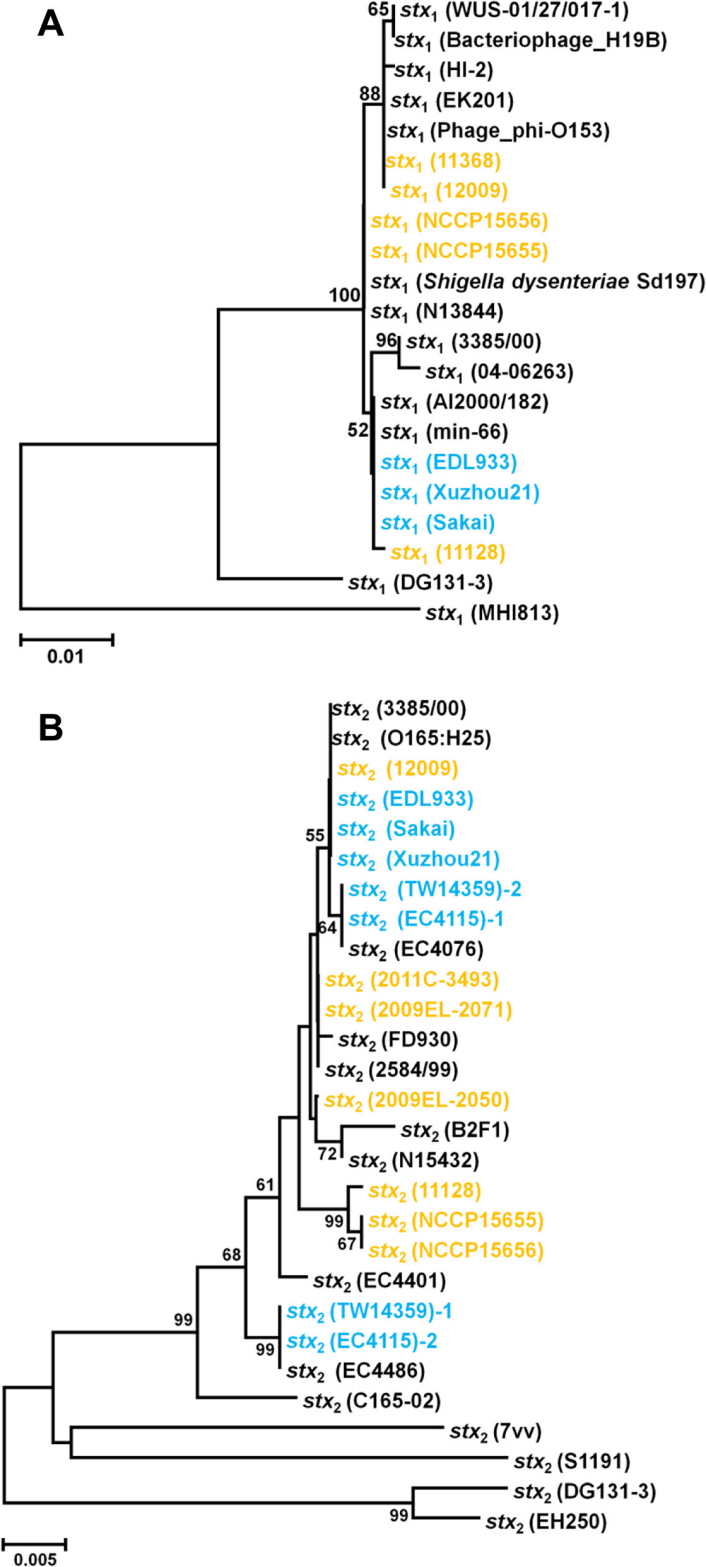
Clustering analysis of the subunit A of the Shiga toxin type 1 and type 2. Un-rooted trees based on the nucleotide sequences of Shiga toxin subunit A were constructed using Neighbor-joining method with Jukes-Cantor model. Bootstrap values (percentages of 1,000 replications) greater than 50% are shown at each node. The scale bar represents 0.005 nucleotide substitutions per site. Yellow, *E. coli* B1 group; Sky blue, *E. coli* E group; Black, unknown **(A)** Shiga toxin type 1, **(B)** Shiga toxin type 2.

In the NCCP15655 and NCCP15656 genomes, genes encoding Shiga toxin type 1 (Stx1) and Shiga toxin type 2 (Stx2) were detected. The Stx1 subunit A is composed of 315 amino-acids and subunit B is composed of 89 amino-acids. In the NCCP15655 genome, the *stx*_1_ genes were detected in the region of a prophage, which have 100% amino-acid identity with the Shiga toxin of *Shigella dysenteriae* Sd197. The Stx2 subunit A is composed of 319 amino-acids and subunit B is composed of 89 amino-acids. The *stx*_2_ genes were detected in another prophage region, which is located at the end of the contig. The *stx*_2_ gene is very similar to that of *E. coli* strain 11128, which has *stx*_1_ genes (Figure 3). The results from subtype analysis of the stx genes indicated that *stx*_1_ is *stx*_1a_ and *stx*_2_ is *stx*_2a_ in both strains. Unlike the typical EHEC strain, in the genomes of NCCP15655 and NCCP15656, the LEE island was not detected but the genes encoding type 1 fimbriae biosynthesis proteins, adhesion AidA, fimbriae-like adhesion SfmA/H, and CFA/I fimbrial minor adhesin were detected. In both strains, a gene encoding type IV pilus biosynthesis proteins, entropathogenic *E. coli* secreted protein C, which is a serine protease and causes epithelial damage [30], and genes encoding hemolysin were detected in the final contigs designated as plasmid and in chromosome, type 1 fimbriae operon were identified.

## Future directions

The Stx phage carrying the Shiga toxin and the LEE island harboring the type III secretion system are the major features of EHEC strains [31]. The genomes of NCCP15655 and NCCP15656 encode the Shiga-like toxin, but not genes related to the LEE island. However, they acquired a plasmid encoding hemolysin and entropathogenic *E. coli* secreted protein C. NCCP15655 and NCCP15656 acquired the virulence genes through the horizontal gene transfer and caused the diarrhea symptom in human. In the case of E24377A, a gene encoding a heat-labile toxin, which is a major virulence factor of ETEC is located in the plasmid but not detected in NCCP15655 and NCCP15656. These mean that, in certain environment, bacterial strains can obtain virulence factors through the acquisition of a virulence gene-harboring plasmid or a phage and cause the disease. This report is yet another example for pathogenic *E. coli* strains that have acquired virulence genes through acquisition of plasmids and phages. These genomes will be good examples for further analysis for the study of acquisition and diffusion of virulence genes in *E. coli*.

## Availability of supporting data

These Whole Genome Shotgun projects of NCCP15655 and NCCP15656 have been deposited at GenBank under the accession ATLW00000000 and ATLX00000000, respectively.

## References

[1] Corrigan JJ, Boineau FG (2001). Hemolytic-uremic syndrome. Pediatr Rev.

[2] Kim BK, Song GC, Hong GH, Seong WK, Kim SY, Jeong H (2012). Genome sequence of the Shiga toxin-producing *Escherichia coli* strain NCCP15657. J Bacteriol.

[3] Song JY, Yoo RH, Jang SY, Seong WK, Kim SY, Jeong H (2012). Genome sequence of enterohemorrhagic *Escherichia coli* NCCP15658. J Bacteriol.

[4] Yatsuyanagi J, Saito S, Ito I (2002). A case of hemolytic-uremic syndrome associated with shiga toxin 2-producing *Escherichia coli* O121 infection caused by drinking water contaminated with bovine feces. Jpn J Infect Dis.

[5] Tarr PI, Neill MA (1996). Perspective: The problem of non-O157:H7 shiga toxin (verocytotoxin)-producing *Escherichia coli*. J Infect Dis.

[6] Menrath A, Wieler LH, Heidemanns K, Semmler T, Fruth A, Kemper N (2010). Shiga toxin producing *Escherichia coli*: identification of non-O157:H7-Super-Shedding cows and related risk factors. Gut Pathog.

[7] Kemper MJ (2012). Outbreak of hemolytic uremic syndrome caused by *E. coli* O104:H4 in Germany: a pediatric perspective. Pediatr Nephrol.

[8] Loos S, Kemper MJ (2012). An Outbreak of Shiga-Toxin Producing *E. coli* O104:H4 Hemolytic Uremic Syndrome (STEC-HUS) in Germany: Presentation and Short Term Outcome in Children. A Report of the German Pediatric HUS Registry. Nephrol Dial Transpl.

[9] Bloch SK, Felczykowska A, Nejman-Falenczyk B (2012). *Escherichia coli* O104:H4 outbreak - have we learnt a lesson from it?. Acta Biochim Pol.

[10] Welinder-Olsson C, Kaijser B (2005). Enterohemorrhagic *Escherichia coli* (EHEC). Scand J Infect Dis.

[11] Bernier C, Gounon P, Le Bouguenec C (2002). Identification of an aggregative adhesion fimbria (AAF) type III-encoding operon in enteroaggregative *Escherichia coli* as a sensitive probe for detecting the AAF-Encoding operon family. Infect Immun.

[12] Ruggenenti P, Remuzzi G (2011). A German outbreak of haemolytic uraemic syndrome. Lancet.

[13] Cho YH, Park HJ, Song KS, Song YG, Lee SI, Park IS (2002). A case of hemolytic uremic syndrome caused by *Escherichia coli* O8: Case Report. Korean J Gastrointest Endosc.

[14] Bae WK, Lee YK, Cho MS, Ma SK, Kim SW, Kim NH (2006). A case of hemolytic uremic syndrome caused by *Escherichia coli* O104:H4. Yonsei Med J.

[15] Jeong H, Zhao F, Igori D, Oh KH, Kim SY, Kang SG (2012). Genome sequence of the hemolytic-uremic syndrome-causing strain *Escherichia coli* NCCP15647. J Bacteriol.

[16] Green MR, Sambrook J (2012). MOLECULAR CLONING A Laboratory Manual.

[17] Boetzer M, Henkel CV, Jansen HJ, Butler D, Pirovano W (2011). Scaffolding pre-assembled contigs using SSPACE. Bioinformatics.

[18] Tsai IJ, Otto TD, Berriman M (2010). Improving draft assemblies by iterative mapping and assembly of short reads to eliminate gaps. Genome Biol.

[19] Salzberg SL, Delcher AL, Kasif S, White O (1998). Microbial gene identification using interpolated Markov models. Nucleic Acids Res.

[20] Vallenet D, Labarre L, Rouy Z, Barbe V, Bocs S, Cruveiller S (2006). MaGe: a microbial genome annotation system supported by synteny results. Nucleic Acids Res.

[21] Aziz RK, Bartels D, Best AA, DeJongh M, Disz T, Edwards RA (2008). The RAST server: Rapid annotations using subsystems technology. BMC Genomics.

[22] Li L, Stoeckert CJ, Roos DS (2003). OrthoMCL: identification of ortholog groups for eukaryotic genomes. Genome Res.

[23] Jeong H, Barbe V, Lee CH, Vallenet D, Yu DS, Choi SH (2009). Genome sequences of *Escherichia coli* B strains REL606 and BL21(DE3). J Mol Biol.

[24] Edgar RC (2004). MUSCLE: multiple sequence alignment with high accuracy and high throughput. Nucleic Acids Res.

[25] Guindon S, Gascuel O (2003). A simple, fast, and accurate algorithm to estimate large phylogenies by maximum likelihood. Syst Biol.

[26] Jones DT, Taylor WR, Thornton JM (1992). The rapid generation of mutation data matrices from protein sequences. Comput Appl Biosci.

[27] Konstantinidis KT, Tiedje JM (2005). Genomic insights that advance the species definition for prokaryotes. Proc Natl Acad Sci U S A.

[28] Richter M, Rossello-Mora R (2009). Shifting the genomic gold standard for the prokaryotic species definition. Proc Natl Acad Sci U S A.

[29] Scheutz F, Teel LD, Beutin L, Pierard D, Buvens G, Karch H (2012). Multicenter evaluation of a sequence-based protocol for subtyping Shiga toxins and standardizing Stx nomenclature. J Clin Microbiol.

[30] Navarro-Garcia F, Canizalez-Roman A, Sui BQ, Nataro JP, Azamar Y (2004). The serine protease motif of EspC from enteropathogenic *Escherichia coli* produces epithelial damage by a mechanism different from that of pet toxin from enteroaggregative *E-coli*. Infect Immun.

[31] Lee JE, Reed J, Shields MS, Spiegel KM, Farrell LD, Sheridan PP (2007). Phylogenetic analysis of Shiga toxin 1 and Shiga toxin 2 genes associated with disease outbreaks. BMC Microbiol.

